# Palbociclib in breast cancer neoadjuvant setting

**DOI:** 10.4322/acr.2021.309

**Published:** 2021-08-20

**Authors:** Sílvia Alexandra Cabral Duarte, Daniela Silva Pádua de Azevedo, Teresa Tomé Ribeiro Malheiro Sarmento, Marta Verónica Velho Sousa

**Affiliations:** 1 Centro Hospitalar de Trás-os-Montes e Alto Douro EPE, Medical Oncology Department, Vila Real, Portugal

**Keywords:** Breast Neoplasms, Cyclin-Dependent Kinase 4, Cyclin-Dependent Kinase 6, Case Reports

## Abstract

Cyclin-dependent kinase 4/6 inhibitors represent a major advance in breast cancer treatment, emerging as the standard of care of the initial treatment of hormone receptor-positive and HER2-negative metastatic breast cancer. Their activity in this subset of patients leads to interest in their use in the adjuvant and neoadjuvant settings. This case report presents a real-life case of cyclin-dependent kinase 4/6 inhibitors use in a patient initially considered to have stage IV luminal HER2-negative breast cancer with liver metastasis. The discrepancy of treatment response between the breast tumor and liver node led to a repetition of the liver biopsy, which revealed metastasis of a neuroendocrine tumor of unknown primary. The breast tumor showed a partial response, and the initial therapeutic strategy was then redefined for curative intent. While cyclin-dependent kinase 4/6 inhibitors are not yet approved for clinical practice in the neo / adjuvant treatment of hormone receptor-positive breast cancer, this case report portrays a successful example of its application in a neoadjuvant setting.

## INTRODUCTION

Cyclin-dependent kinase (CDK) 4 and 6 inhibitors act by inhibiting progression from the G1 to S phases of the cell cycle. Their approval in postmenopausal women with metastatic hormone receptor-positive, human epidermal growth factor receptor (HER) 2-negative breast cancer represents a breakthrough in cancer therapeutics. It is well documented that CDK 4/6 inhibitors, palbociclib, ribociclib and abemaciclib, in association with endocrine therapy significantly improve progression-free and overall survival in the context of metastatic luminal breast cancer. 3

The benefits demonstrated by CDK 4/6 inhibitors in the metastatic setting led to an interest in their use in the curative setting as well. Several concluded and ongoing clinical trials, discussed further in the discussion section, evaluate the effectiveness of CDK inhibitors 4/6 in adjuvant and neoadjuvant treatment of localized breast cancer with encouraging preliminary results. 5 ^–^ 12

This clinical case illustrates the unintended use of CDK4/6 inhibitors in the context of neoadjuvant treatment of hormone receptor-positive, HER2-negative breast cancer. In fact, it is a real-life case where CDK4/6 inhibitors plus aromatase inhibitors were successful in obtaining a partial imaging response in neoadjuvant treatment of early breast cancer, which was initially assumed as metastatic, while this strategy remains investigational.

## CASE REPORT

A 42-year-old female patient with an ECOG-PS 0 was referred to the senology consultation by her attending physician due to a mammography classified as BIRADS 5 performed in the national breast cancer screening program. Her medical history was notable for generalized anxiety disorder, medicated with alprazolam 1mg id, sertraline 50mg id, trazodone 50mg id, without any other relevant medical or surgical comorbidities. She did not consume alcohol or tobacco, had a sedentary lifestyle and a balanced and diverse diet. Regarding her gynecological history, menarche occurred at 16 years old. She had one gestation (eutocic delivery) and zero abortions and breastfed for six months. She denied the use of oral contraceptives and was pre-menopausal. She had no family history of oncologic disease in relatives of 1st and 2nd degree. Her physical examination was remarkable for a hard and painless breast lump of approximately 4 cm in the upper outer quadrant of the left breast, without palpable axillary or supraclavicular adenomegaly. The remaining physical examination was unremarkable.

**Figure 1:**
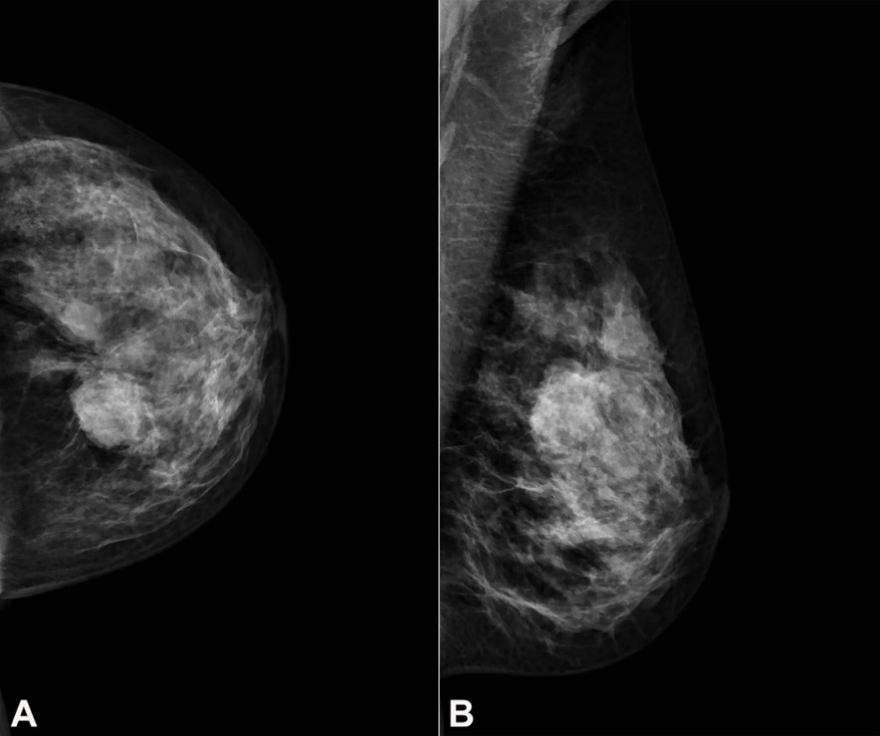
**A** – Mammography in craniocaudal; and **B** – mediolateral oblique views, showing a solid irregular lesion with 50x26 mm in the upper outer quadrant of the left breast, suspicious for malignancy.

The mammography described a heterogeneously dense breast pattern with a solid irregular lesion with 50x26 mm in the upper outer quadrant of the left breast (Figure 1A, B).

**Figure 2:**
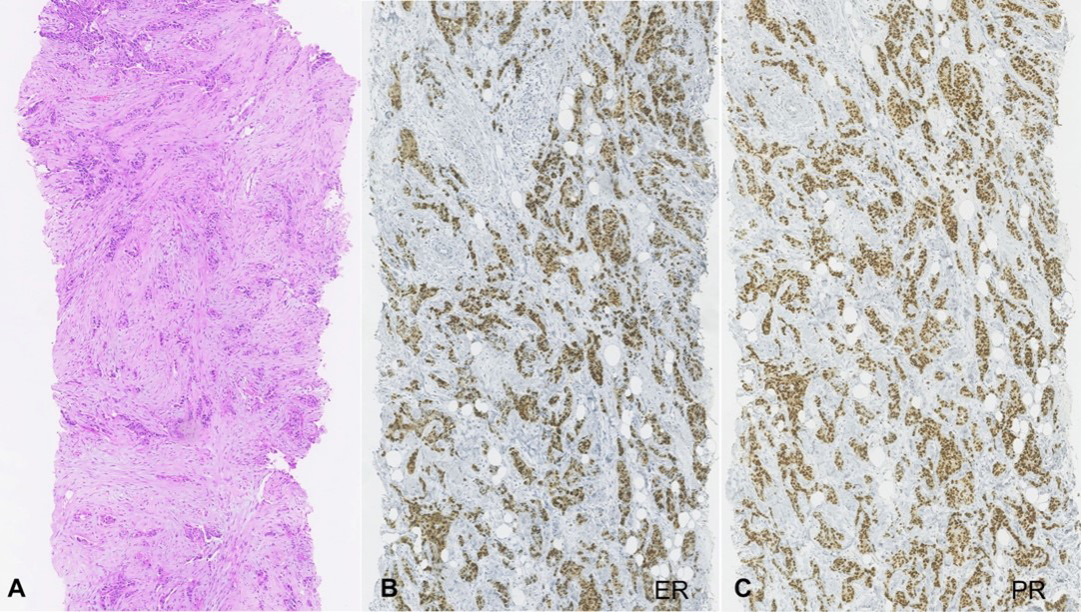
Photomicrograph of the microbiopsy of the left breast lesion consistent with: **A** – Invasive carcinoma with 60% of ducts, high nuclear pleomorphism, 4-5 mitoses for 10 high power fields, with no evidence of vascular or perineural infiltration. Images of ductal carcinoma in situ with high nuclear grade and comedocarcinoma. Histology compatible with the diagnosis of invasive ductal carcinoma (H&E x40); **B** – immunohistochemistry positivity for estrogen-receptor expression (x100); **C** – progesterone-receptor positive expression (x100).

A microbiopsy of the left breast lesion revealed an invasive breast carcinoma of the ductal type with the following immunohistochemistry profile: estrogen-receptor expression (ER) of 70-80%; progesterone-receptor expression (PR) of 40-50%; HER-2 negative; Ki67 proliferation index of 40% (Figure 2).

A staging thoracoabdominopelvic computed tomography was performed, which revealed no mediastinal, hilar, or axillary adenomegaly, but the presence of a 10 mm hepatic nodule with features suggestive of hepatic metastasis and a 6 mm bone nodule in the right iliac wing with non-specific features. The bone scintigraphy was performed for clarification, showing no hyper fixation in the referred bone nodule. Also, an abdominal MRI was performed for better imaging characterization, showing a 21 mm lesion of heterogeneous content and exophytic growth in the left lobe, and several small hypodense subcapsular nodules (maximum diameter 10 mm) in hepatic segments VII and VIII, consistent with hepatic micro metastases. A complementary PET-Scan evaluation was performed, showing 18-FDG hypermetabolism of the upper outer quadrant of the left breast, compatible with known breast cancer, and absence of hyper capture in the hepatic parenchyma.

A hepatic biopsy was performed being compatible with metastasis of carcinoma with tubular differentiation. The immunohistochemistry evaluation was pending on that date. With the final diagnosis of invasive ductal carcinoma of the breast, staged as cT3N0M1 (liver metastasis), the patient initiated palbociclib (125mg, cycles of 28 days, 21 days on – 7 days off) in association with letrozole (2.5 mg once daily) plus goserelin (3.6 mg every 28 days).

**Figure 3:**
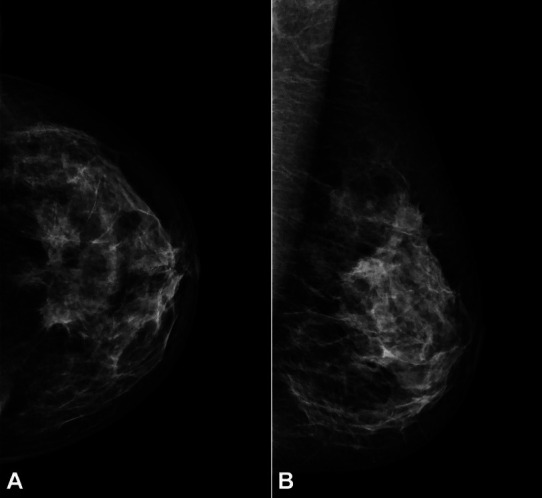
**A** – Mammography in craniocaudal; **B** – and mediolateral oblique views, showing partial response to palbociclib in association with letrozole plus goserelin.

After completion of 3 cycles, a bilateral mammogram and an abdominal MRI for imaging response assessment were performed. Mammography showed a nodular lesion in the upper outer quadrant of the left breast consistent with the previously identified tumor. A dimension reduction to 31x10 mm (previous: 50x26 mm) was observed, corresponding to a partial response by RECIST 1.1 Criteria^1^ (Figure 3).

On the other hand, the abdominal MRI revealed an increased dimension of the exophytic subcapsular nodule in the left lobe to 33x22mm, being the previous largest diameter of 21 mm.

**Figure 4:**
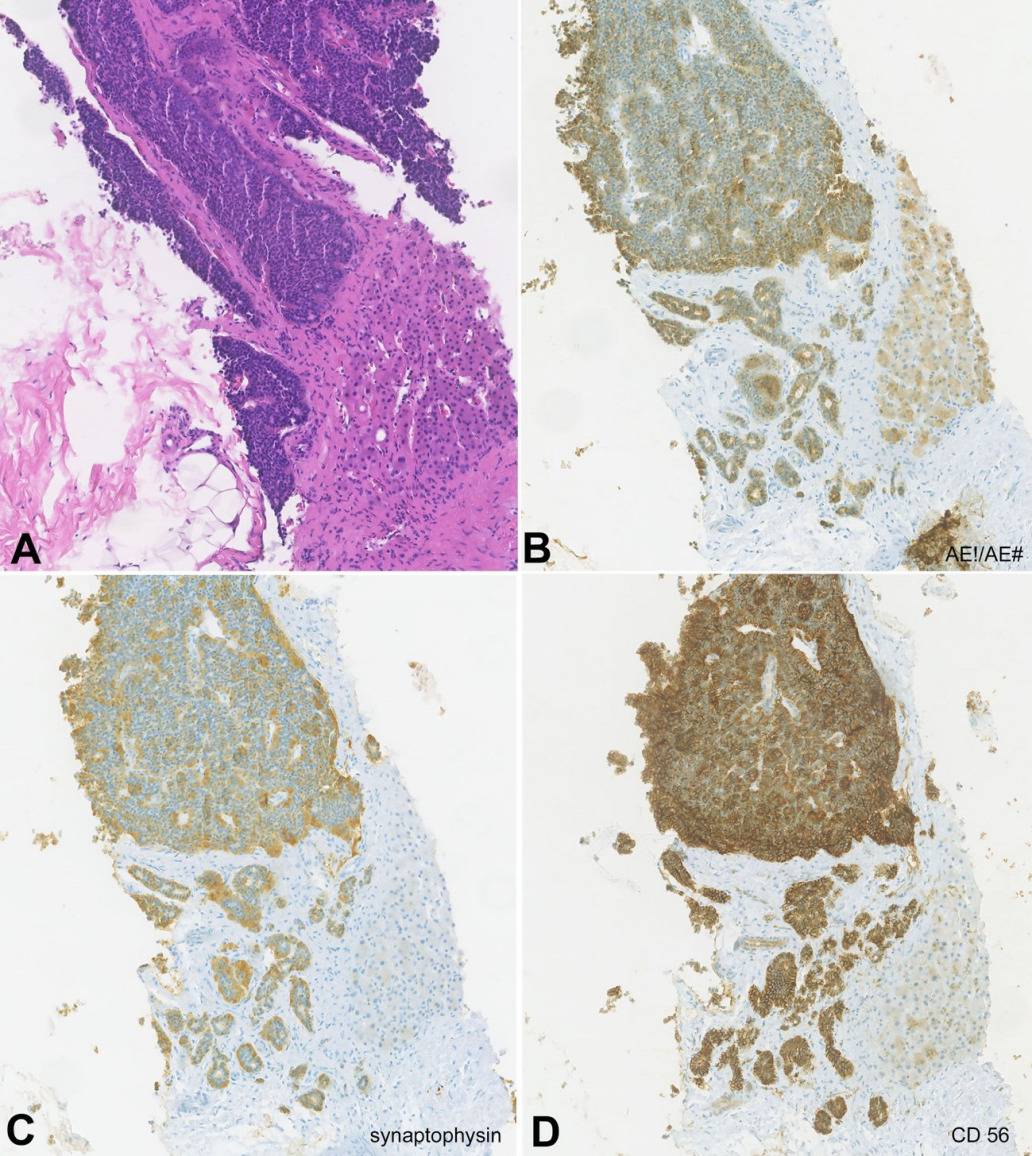
Photomicrographs of liver biopsy showing: **A** – hepatic parenchyma occupied by well-differentiated neuroendocrine tumor (H&E x100); immunohistochemistry analysis showing positivity for **B** – AE1 / AE3, a broad-spectrum cytokeratin positive in epithelial neoplasms (x100); **C** and **D** – positivity for synaptophysin and CD56, both neuroendocrine markers (x100).

**Figure 5:**
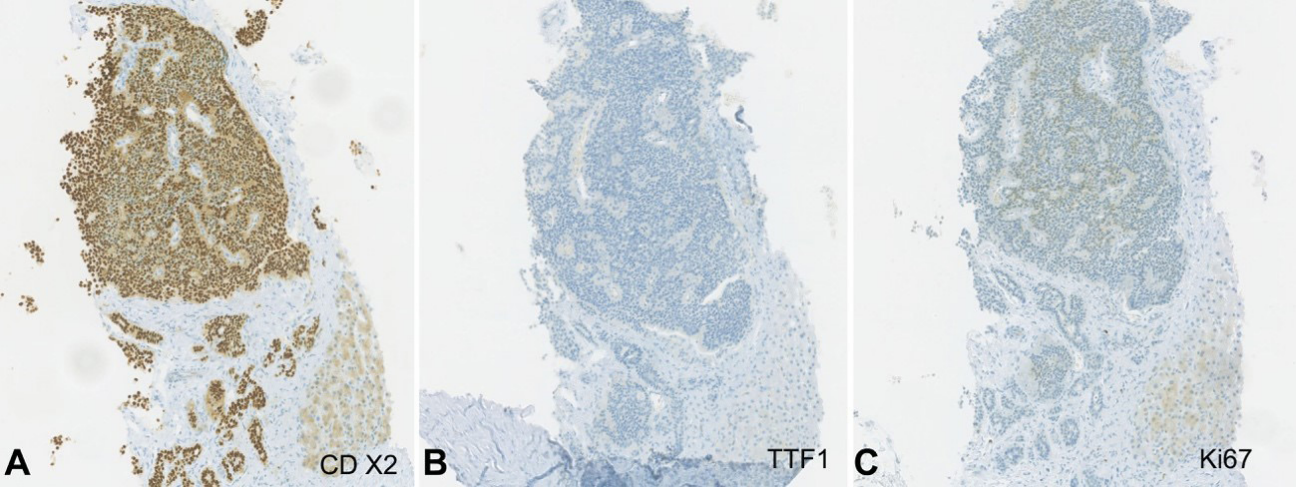
Photomicrographs of liver biopsy showing: **A** – positivity for CDX2, a specific marker of intestinal epithelial cells (x100); **B** – negativity for TTF1, a marker typically negative in extrapulmonary neuroendocrine tumors (x100); **C** – Less than 1% of the tumor cell population was positive for Ki67 expression (x100).

The discrepancy between the response of the breast tumor and liver node led to a repetition of the liver biopsy​. Histological examination showed hepatic parenchyma almost entirely occupied by well-differentiated neuroendocrine tumor metastasis growing in irregular solid cell nests, without areas of visible necrosis or mitosis. The immunohistochemistry evaluation was positive for diffuse expression of pancitokeratins (AE1 / AE3), synaptophysin, CD56, and CDX2 and negative for TTF1. Less than 1% of the tumor cell population was positive for Ki67 expression. The observed immunophenotypic profile was suggestive of a primary tumor of the digestive tract, namely the ileum and ileocecal appendix. The immunohistochemistry of the first liver biopsy was reviewed and was also compatible with hepatic metastasis of a neuroendocrine tumor (Figures 4 and 5).

**Figure 6:**
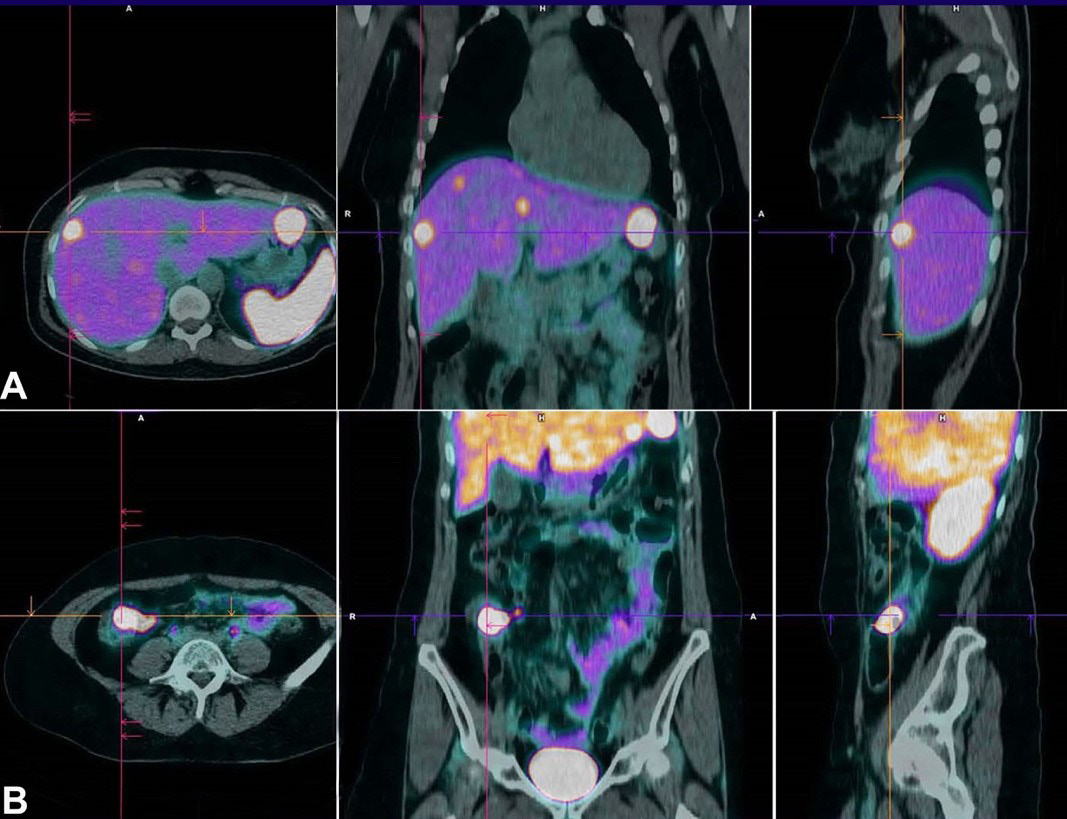
PET 68Ga-DOTANOC showing **A** – liver metastases; and **B** – a primary tumor in the ileocecal topography with high expression of somatostatin receptors.

A PET 68Ga-DOTANOC was performed to determine the location of the primary neuroendocrine tumor as well as the extension of the disease, and it was conclusive for liver metastases and a primary tumor in the ileocecal topography with high expression of somatostatin receptors (Figure 6).

Therefore, the patient was finally diagnosed with invasive ductal carcinoma of the breast cT3N0M0 and a low-grade neuroendocrine tumor​ cTxNxM1. After discussion in a multidisciplinary cancer team, the patient suspended palbociclib, maintained letrozole plus goserelin and was proposed for conservative breast surgery with sentinel lymph node biopsy, with curative intent. As a palliative strategy for the treatment of neuroendocrine tumor, the patient initiated an analog of somatostatin: lanreotide 120 mg subcutaneous 4/4 week. The breast surgical specimen revealed an invasive ductal carcinoma of 2mm and several scattered foci of DCIS high nuclear grade. The immunohistochemistry evaluation was compatible with a molecular subtype luminal B HER-2 negative (ER expression of 70-80%; PR expression of 40-50%; HER-2 negative; Ki67 of 40%) ​ invasive ductal carcinoma, grade 4 of Miller-Payne histological response,^2^ and the sentinel lymph node biopsy was negative for neoplasia. The final stage was ypT1aN0M0​. The patient was proposed for a prognostic gene expression assay, OncotypeDX©, that help assess the risk of breast cancer recurrence and predict chemotherapy benefit through analysis of a set of cancer-related genes in patient's breast tumor tissue. The test scored 49/100, equivalent to a 38% probability of recurrence with hormone therapy at 9 years and a chemotherapy benefit superior to 15%. Consequently, the patient was proposed for adjuvant chemotherapy (4 cycles of docetaxel 75 mg/m2 plus cyclophosphamide 600 mg/m2) followed by radiotherapy and endocrine therapy with letrozole 2.5 mg daily plus goserelin​ 3.6 mg subcutaneous 4/4 week and maintenance of lanreotide.

To date, and after 3 years since diagnosis, the patient has an ECOG – Performance Status of 0 with good tolerance to ongoing treatments, without evidence of breast cancer recurrence, and with a dimensional and numeric stability of the metastatic neuroendocrine tumor.

## DISCUSSION

The CDK 4/6 pathway is overactive in several cancers, including breast cancer, and its inhibition leads to cell cycle arrest. In postmenopausal women with hormone receptor-positive metastatic breast cancer, the combination of letrozole with CDK 4/6 inhibitors (palbociclib, ribociclib, or abemaciclib) have demonstrated improvement in progression-free survival comparative to an aromatase inhibitor alone, which led to its approval by the United States Food and Drug Administration and the European Medicines Agency in this setting.^3^

Currently, several aspects regarding the use of CDK4/6 inhibitors are being explored such as, in adjuvant and neoadjuvant settings in localized breast cancer treatment, as well as in association with immunotherapy, since CDK4/6 inhibitors exposure might enhance the response to immunotherapy agents.^4^

Hormone receptor-positive, HER2-negative metastatic breast cancer typically presents as a late recurrent disease. Hence, exploring new strategies that aim to reduce recurrence risk is crucial in the perspective of a curative treatment.

Clinical trials in the neoadjuvant setting, have shown that CDK4/6 inhibitors significantly increase cell-cycle arrest compared with hormone therapy alone. The phase 2 neoadjuvant NeoPalAna study 5 showed that the addition of palbociclib to anastrozole caused enhanced antiproliferative activity, with 87% complete cell-cycle arrest compared to anastrozole alone with a 26% complete arrest. In the NeoMONARCH study,^6^ the addition of abemaciclib to anastrozole in previously untreated postmenopausal women with early-stage HR-positive, HER2-negative breast cancer, also showed to significantly reduce Ki67 compared with anastrozole alone (66% versus 15%, respectively). Similar results for palbociclib were observed in the PALLET study.^7^ Curigliano et al. 8 assessed the biological activity of ribociclib with letrozole compared to letrozole alone as neoadjuvant therapy and, in the arm treated with ribociclib plus letrozole, a decrease in the percentage of Ki67 positive cells of 92% was observed, versus 69% in the letrozole-alone arm. Neoadjuvant trials comparing endocrine therapy and CDK 4/6 inhibitor versus chemotherapy have shown equivalent outcomes in favor of CDK 4/6 inhibitor.^9^^,^ 10 However, further investigations are required since it is unknown whether Ki67 reduction directly translates into long-term clinical benefit. FELINE study (NCT02712723), a phase 2 ongoing clinical trial, will help clarify the potential role of CDK4/6 inhibitors in neoadjuvance. The main purpose of this study is to determine if ribociclib in combination with letrozole for 24 weeks in a neoadjuvant setting increases the proportion of women with a Pre-operative Endocrine Prognostic Index (PEPI) score of 0 at surgery compared to patients treated with letrozole alone.

In this case, the patient achieved a partial response in breast cancer with palbociclib in association with letrozole, and not a complete response which is an established prognostic factor and a surrogate endpoint. The phase 2 trails described above use surrogate endpoints instead of clinical endpoints, such as disease-free survival or overall survival. Therefore, the available evidence is not yet robust enough to support the current use of CDK4/6 inhibitors in the neoadjuvant setting. It is noteworthy that, subsequently, it was realized that the patient also presented a high score on Oncotype DX©, which favors chemotherapy use. Still, a partial pathological response was observed with palbociclib plus letrozole.

In the adjuvant treatment, preliminary results of two recent trials were promising, despite the uncertain data about the role of CDK4/6 inhibitors in this setting. The full report of these trials, including overall survival results, is still awaited. In the monarchE trial, among over 5600 patients with high risk, hormone receptor-positive, HER2-negative early breast cancer, the addition of abemaciclib to endocrine therapy resulted in absolute improvements in invasive disease-free and distant relapse-free survival by approximately 3 to 4 percent at two years.^11^ However, in preliminary results of the PALLAS trial, the addition of palbociclib to adjuvant endocrine therapy failed to improve outcomes.^12^

At the moment, the neoadjuvant treatment in hormone receptor-positive, HER2-negative breast cancer is based on chemotherapy and is recommended in selected cases such as inflammatory breast cancer, bulky N2 axillary nodes, N3 nodal disease, T4 tumors, and large primary tumor relative to breast size in a patient who desires breast conservation. For these patients, it might be a valid option in the future to use CDK4/6 inhibitors instead of chemotherapy since it allows to decrease related toxicities and reduce hospital visits for a similar potential benefit.

There are also studies in HER2-positive breast cancer and triple-negative breast cancer with interesting results but without potential application in clinical practice. 4 ^,^ 13 ^,^ 14

Pending further data, the use of CDK 4/6 inhibitors in the neoadjuvant and adjuvant setting remains investigational.

## CONCLUSION

In view of the unequivocal benefits of CDK4/6 inhibitors in association with endocrine therapy in the metastatic setting for patients with hormone receptor-positive, HER2-negative breast cancer, it is of interest to explore these drugs in curative settings, namely in the neoadjuvant and adjuvant treatment of localized breast cancer. Further data from the ongoing clinical trials are needed to expand and refine the clinical application of CDK4/6 inhibitors in patients with breast cancer.

It is also worth mentioning the relevance of the histological diagnosis of suspected metastatic lesions of known primary tumors. The possibility of two different primary tumors is not negligible under these circumstances and may lead to inaccurately therapeutic decisions.

## References

[1] Litière S, Isaac G, De Vries EG (2019). RECIST 1.1 for response evaluation apply not only to chemotherapy-treated patients but also to targeted cancer agents: a pooled database analysis. J Clin Oncol.

[2] Ogston KN, Miller ID, Payne S (2003). A new histological grading system to assess response of breast cancers to primary chemotherapy: prognostic significance and survival. Breast.

[3] Gao JJ, Cheng J, Bloomquist E (2020). CDK4/6 inhibitor treatment for patients with hormone receptor-positive, HER2-negative, advanced or metastatic breast cancer: a US Food and Drug Administration pooled analysis. Lancet Oncol.

[4] He S, Roberts PJ, Sorrentino JA (2017). Transient CDK4/6 inhibition protects hematopoietic stem cells from chemotherapy-induced exhaustion. Sci Transl Med.

[5] Ma CX, Gao F, Luo J (2017). NeoPalAna: neoadjuvant palbociclib, a cyclin-dependent kinase 4/6 inhibitor, and anastrozole for clinical stage 2 or 3 estrogen receptor–positive breast cancer. Clin Cancer Res.

[6] Martin M, Hurvitz SA, Chan D, American Association for Cancer Research (2018). Final results of NeoMONARCH: a phase 2 neoadjuvant study of abemaciclib in postmenopausal women with hormone receptor positive (HR+), HER2 negative breast cancer (BC). Cancer research.

[7] Johnston S, Puhalla S, Wheatley D, Ring A, Barry P, Holcombe C ( 2019). Randomized Phase II study evaluating palbociclib in addition to letrozole as neoadjuvant therapy in estrogen receptor-positive early breast cancer: PALLET Trial. Am J Clin Oncol.

[8] Curigliano G, Pardo PG, Meric-Bernstam F (2016). Ribociclib plus letrozole in early breast cancer: a presurgical, window-of-opportunity study. Breast.

[9] Cottu P, D’Hondt V, Dureau S (2018). Letrozole and palbociclib versus chemotherapy as neoadjuvant therapy of high-risk luminal breast cancer. Ann Oncol.

[10] Prat A, Saura C, Pascual T (2020). Ribociclib plus letrozole versus chemotherapy for postmenopausal women with hormone receptor-positive, HER2-negative, luminal B breast cancer (CORALLEEN): an open-label, multicentre, randomised, phase 2 trial. Lancet Oncol.

[11] Johnston SR, Harbeck N, Hegg R, Toi M, Martin M, Shao ZM (2020). Abemaciclib combined with endocrine therapy for the adjuvant treatment of hr+, her2-, node-positive, high-risk, early breast cancer (monarche). J Clin Oncol.

[12] Mayer E, Gnant M, DeMichele A (2020). LBA12 Pallas: a randomized phase III trial of adjuvant palbociclib with endocrine therapy versus endocrine therapy alone for HR+/HER2-early breast cancer. Ann Oncol.

[13] Bhattacharya K, Yang Y (2016). A cost-effectiveness analysis of palbociclib and other aromatase inhibitors for treatment of advanced breast cancer. Value Health.

[14] Matter-Walstra K, Ruhstaller T, Klingbiel D, Schwenkglenks M, Dedes K (2016). Palbociclib as a first-line treatment in oestrogen receptor-positive, HER2-negative, advanced breast cancer not cost-effective with current pricing: a health economic analysis of the Swiss Group for Clinical Cancer Research (SAKK). Breast Cancer Res Treat.

